# Driving mechanism of consumer migration behavior under the COVID-19 pandemic

**DOI:** 10.3389/fpubh.2022.1005265

**Published:** 2023-01-05

**Authors:** Dong Wang, Weishan Chen, Xiarou Zheng, Xuetong Wang

**Affiliations:** School of Management, Guangzhou University, Guangzhou, China

**Keywords:** normalized epidemic prevention and control, channel integration, perceived value, channel migration intention, VAM model

## Abstract

**Introduction:**

China is now in the post-period of COVID-19 epidemic prevention and control. While facing normalized epidemic prevention and control, consumers behavioral intention and decision-making will still be influenced by the epidemic's development and the implementation of specific epidemic prevention measures in the medium to long term. With the impact of external epidemic prevention environment and measures, consumers' channel behavior has changed. How to better promote channel integration by adopting consumers' channel migration behavior is important for channel coordination strategies.

**Methods:**

This paper takes fresh product retailing under normal epidemic prevention and control as an example and examines the change in channel migration behavior. Based on the value-based adoption model (VAM), this paper discusses the influence of channel characteristics and channel switching costs on channel migration intention, the mediating effect of perceived value between various influencing factors and channel migration intention, and the moderating effect of channel switching cost on perceived value and channel migration intention. Thus, an empirical study was carried out with 292 samples to verify the hypotheses.

**Results:**

The results show that under normal epidemic prevention and control, the influencing factors in the VAM model have a significant impact on channel migration intention; perceived value plays a mediating role between various influencing factors and channel migration intention.

**Discussions:**

The COVID-19 pandemic has had a significant effect on daily life and purchasing behavior. In the context of this pandemic, we have confirmed that consumers will probably change to other retailers when the usefulness, entertainment, and cost meet their expectation for purchasing fresh products. Channel characteristics have versatile features, such as channel structure and supply chain mode, which affect consumer behaviors in different ways. The perceived value comes from expectations and experience. Retailers should try to keep their products fresh and provide consumers with a high-level shopping experience during sale.

## 1. Introduction

According to the Regulations on Emergency Response to Public Health Emergencies, public health emergencies refer to major infectious diseases, mass unexplained diseases, major food and occupational poisoning, and other events that seriously affect public health that suddenly occur and cause or may cause serious damage to public health (1–3). Since modern times, there have been many serious public health events at home and abroad, such as mad cow disease that broke out and spread widely in Britain and Europe in 1986, carcinogen contamination in Belgium in 1999, foot-and-mouth disease in Britain and Europe in 2001, the melamine-tainted milk powder incident in 2008, and the national SARS infection in 2013. Since 2020, COVID-19 has ravaged the world, and the World Health Organization (WHO) has listed the COVID-19 epidemic as a public health emergency. Its virus spreads rapidly *via* transmission modes such as droplet transmission, contact transmission, and aerosol transmission, which makes the epidemic difficult to prevent and control, and it has a wide range of infection. Because of its serious harm, China quickly started its first-level response to major public health emergencies. Although the national government has launched effective defense measures, the virus cannot be completely isolated, and powerful treatment measures still need to be developed. The repeated epidemics have kept the whole country in a long-term epidemic prevention state. Under the background of normalized epidemic prevention measures, residents' lives, work, and travel are limited, and residents' psychology and actions have undergone profound changes (4, 5).

Under the COVID-19 pandemic, consumers' behavior is more rational and cautious, and residents tend to consume more safely, healthily, conveniently, and efficiently (6–8). To meet actual needs, retailing channels have been continuously developed and improved. In offline channels, the government has proposed new regulations for entering public shopping spaces, such as checking body temperature, wearing masks, and presenting health QR codes or nucleic acid test certificates, which control the number of offline consumers. Thus, most merchants have developed online trading platforms, support contactless distribution, and provide zero-contact services, such as intelligent robots and self-service payment machines. Community consumption, community group purchase, and other last-mile consumption scenarios and consumption patterns are being innovated and continuously derived. Shopping through online channels is convenient and fast, and because the cost of operating online is lower than for offline stores, the product price is relatively low. However, the experience provided by offline channels to consumers is markedly different from online shopping, and each type of channel has its own advantages and disadvantages (6, 9, 10). Compared with the steady growth trend in previous years, total consumption in 2020 showed a certain degree of decline due to the epidemic situation. From the perspective of specific consumption categories, the per capita consumption expenditure on food, tobacco, and alcohol increased by 4%; the per capita consumption expenditure on education, culture, and entertainment decreased by 2.1%; and the per capita consumption expenditure on clothing, daily necessities and services, and transportation and communication showed either a small increase or decrease.^1^ Enhancement of the shopping experience has been addressed by previous studies, and new retailing modes combining online and offline have become an urgent concern for retailers (11–13). An increasing number of retail enterprises have begun to use channel layouts to further improve the purchasing efficiency for customers and improve the satisfaction of customers' purchasing experience (14–17).

Moreover, with the rapid development of internet information technology, the number of mobile network users in China is increasing, which has caused rapid changes in people's work and lifestyle, and consumers' perceived value of different channels is also different, which will prompt consumers to make different channel selection decisions (18–21). Therefore, a group of consumers who prefer using online channels for shopping has emerged that attaches great importance to the convenience of purchasing methods. It is difficult to meet the needs of this consumer group by relying only on a single purchase channel in the shopping process, and the behavior of dynamically switching shopping channels in the consumption process (i.e., channel migration behavior) is becoming increasingly common.

To address these issues in the context of the COVID-19 pandemic, this paper proposes the following research questions:

(1) How is consumer migration behavior changing?
(2) How does the COVID-19 pandemic affect consumer migration behavior?
(3) What are the key influencing factors in consumer migration behavior? What is the driving mechanism behind consumer migration behavior?

This paper contributes to both the theoretical and practical spheres by answering these questions. First, previous studies have mainly focused on crossing behavior from online to offline, or vice versa, but research on attitudes toward fresh products is limited (10, 22). We investigate consumer's channel behavior regarding fresh product purchasing during the pandemic by using Hema Fresh retailing as an example. Interviewing Hema Fresh's members, information on purchase behaviors and intentions was collected using structured questions. Thirty-one records were analyzed to summarize the characteristics of migration behaviors and the potential affecting factors. We noted that the characteristics of retailing channels mediate consumers' migration behaviors.

The second contribution of this paper is to identify the influencing factors and propose measurements of these latent variables. Previous research has shown that channel structures and consumers' perceived value, perceived usefulness, and perceived entertainment of purchasing channels affect consumers' channel choices (5, 23–26). Through interviewing Hema Fresh's members, they demonstrated that the experience (e.g., delivery time, freshness, payment convenience) will significantly affect migration behavior. Based on the results of the investigation, nearly 90% of consumers would change to other retailers if the experience is not good with Hema Fresh. We thus measured the experience using perceived usefulness, perceived entertainment, and perceived cost.

The third contribution is to propose the driving mechanism behind consumer migration behavior based on the value-based adoption model (VAM). Compared with previous studies, in our model, we set switching cost as a mediating factor in perceived value and cross-channel migration intention. Furthermore, the results show that the mediating effect of channel switching cost on perceived value and channel migration intention is not significant. This is because channel migration behavior is distinct from cross-channel retention intention. The premise of cross-channel retention intention is that consumers choose the same retailer when dynamically changing channels in the stages of information collection and purchase. Retailers should create strategies for enhancing the shopping experience to promote the sale of fresh products.

This paper is organized as follows. We review the literature in section 2. In section 3, research hypotheses and the research model are proposed based on previous studies, and measurements are proposed for the latent variables. In section 4, we present the collected data and analyze it according to the proposed hypotheses, and the hypotheses are checked. We conclude the paper with a discussion.

## 2. Literature review

### 2.1. Impact of public health safety consumption

Consumers are paying more attention to the safety of their environment, food, and residence. Particularly after events involving African swine fever and SARS, the outbreak of the COVID-19 pandemic has seriously threatened the survival and development of residents. Under the background of normalized epidemic prevention, it will affect residents' consumption behavior in the future. Research on the impact of major public health emergencies on residents' consumption behavior decision-making has become a hot issue in academia (1, 2).

#### 2.1.1. The influence of the public health safety consumption market

With the occurrence of major public health emergencies, problems have been identified by macroeconomic research, such as increased uncertainty, short-term consumption degradation, and blockade policies leading to lower consumption expenditure and employment difficulties (1). Cocco and De-Juan-Vigaray (27) studied the causal relationship between a local blockade and consumer spending and employment under the epidemic situation using questionnaire survey data; a blockade not only has a negative impact on employment but also greatly affects families' expectations for the economy. Hu et al. (28) found that consumption upgrading and downgrading coexist under the epidemic situation by analyzing the current situation of urban and rural residents' consumption demand. Affected by the epidemic situation, short-term consumption downgrading becomes a deterministic event. Further research shows that consumption intention plays an inhibitory role in urban and rural residents' consumption behavior in the long run, and inhibition is greater among urban residents than rural residents. Residential consumption has a crowding-out effect on urban and rural residents, and housing, education and culture, transportation and communication, and medical care may become consumption hotspots after the epidemic. However, there are both dangers and opportunities due to the epidemic. Flavián et al. (29) predicted the impact of the epidemic on digital consumption and analyzed the release of improved consumption demand, the mainstream mode of online consumption, the multiscenario application of unmanned equipment, and the vigorous rise of community commerce. Haider et al. (22) believed that the epidemic has created new scenes for digital technology innovation breakthroughs, opened up new spaces for industrial digital integration and development, and provided new energy for the rise of digital consumption.

#### 2.1.2. Influence of public health safety consumption behavior

Much research has been conducted on the decision-making behind residents' consumption behavior due to major public health emergencies. Particularly since the outbreak of COVID-19, research on the influence of epidemic situations on the decision-making behind consumption behavior has mushroomed and continues to increase (30). Rawat et al. (31) summarized the impact of the COVID-19 epidemic on people's lifestyle behavior according to survey data from residents. Their analysis showed that there were significant changes in residents' diet or eating behavior, stress and mood, sleep style, and physical activity level. The severity of the COVID-19 epidemic is more likely to lead to irrational consumption behavior among consumers, and the demand due to herd consumption, impulsive consumption, and scarce consumption increases (32). In addition, others have discussed the impact of the epidemic from more detailed aspects, such as consumers' choice of commodity types, packaging, and consumption scenarios and methods. For example, Byrd et al. (33) stated that consumers may think that goods (or food) are exposed to COVID-19, which leads to exposure to dangerous sources when buying or eating out, thus reducing consumption or dining out (34). Using a closed Liken scale, data on 1,700 customers from different dimensions were collected, and AMOS software analyzed the data using structural equation models, showing that products related to hygiene, basic food, and home entertainment were becoming more important in shopping lists. At the same time, the socio-economic scene has changed the way consumers buy. Baker et al. (35) used microdata to study the impact of the epidemic on consumer spending. Their results showed that in the first half of March 2020, the total expenditure of individuals increased by more than 40%. Following that, overall spending fell 25%−30% in the second half of March, but spending on food and groceries rose.

Thus, the change in consumer migration behavior during the COVID-19 pandemic has seldom been addressed. It is necessary to study the effect of experience on consumer migration behavior during the pandemic.

### 2.2. Consumer channel migration behavior

The technology acceptance model (TAM) and VAM are applied in examining consumer channel migration behavior.

#### 2.2.1. Consumer channel migration behavior in TAM

**Figure 1:**
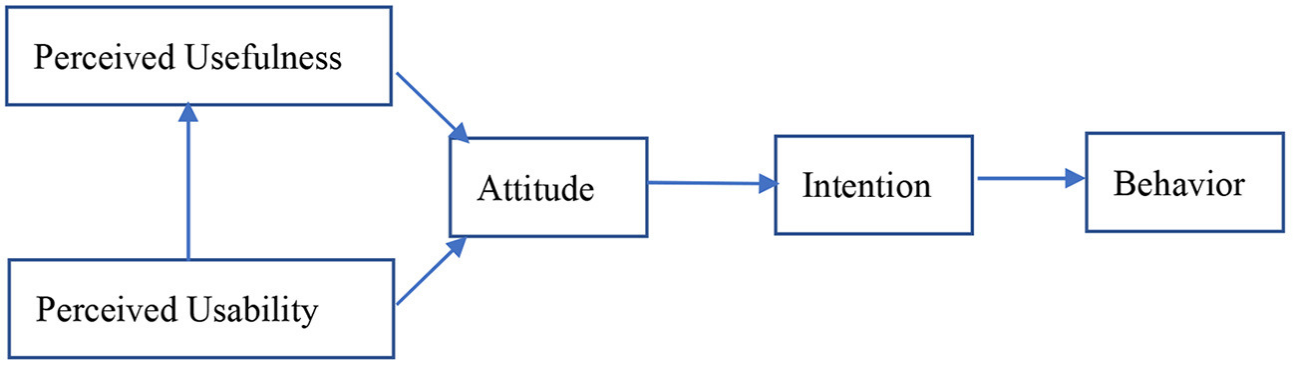
Technology acceptance model (37).

TAM has been put forward in the research field of information system acceptance as a means to predict users' intention and behavior (36). The model is shown in Figure 1.

In the TAM model, the first thing users perceive is a system's usefulness and ease of use, and these comprehensive perceptions have a direct effect on users' attitude. The feelings that users have about a system will affect their intention and behavior. At the same time, users' perceived usability also has an impact on perceived usefulness; that is, the easier the system is to use, the more users will think the system is useful. In addition, external variables, such as demographic factors, will also affect perceived usefulness and perceived ease of use.

**Figure 2:**
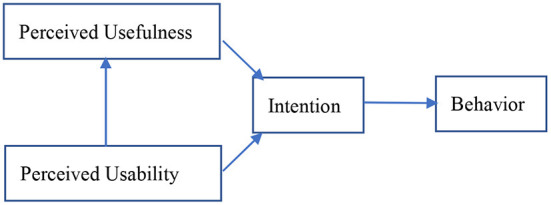
New technology acceptance model.

Previous studies have suggested that behavioral attitude as a moderating variable between perception and intention to use is inappropriate and cannot achieve the purpose of explanation; (38–40) thus, an improved TAM model is proposed. The attitude variable was removed from the new TAM model, as shown in Figure 2.

Many studies have adopted TAM for the study of fresh product retail, particularly for the study of online buying behavior. Acquila-Natale et al. (41) assessed indicators of channel integration and digital transformation of large clothing and apparel retailers in Spain before and after COVID-19 and analyzed the differences by means of McNemar's test and one-way repeated-measures ANOVA. The analysis suggested that large retailers were moderately prepared for providing multichannel and omnichannel services and that they focused on integrating quick and easy-to-implement services. Cocco and De-Juan-Vigaray (27) developed a typology of omnichannel retailer activities and corresponding customer responses during the rapidly changing COVID-19 pandemic to contribute to academic research on omnichannel strategies and to assist retailers when making future investment and resource decisions.

Radhi and Zhang (42) studied and compared four different return policies under which a dual-channel retailer offers both same- and cross-channel returns. For each return policy, they proposed mathematical models to determine optimal order quantities that account for the impact of resalable returns. Van Baal and Dach (43) looked at the purchase of fresh agricultural products and found that consumers' perception of online channels as useful and easy to use had a positive impact on consumers' intention to migrate to online channels for purchase.

Previous studies have provided in-depth research on the influencing factors of channel migration intention, with the main influencing factors mainly coming from the consumer dimension, product dimension, channel dimension, and situation dimension. Previous studies have mainly studied the influence of a single factor or the interaction of multiple factors; select studies are summarized in Table 1.

**Table 1:** Influencing factors of channel migration intention.

**Scholar (year)**	**Factors**	**Content**
Gupta et al. (38), Trampe et al. (39)	Consumers	Perceived risk, self-efficacy, consumer trust and so on
Baker et al. (35)	Products	Price, experience, brand marketing, etc.
Li et al. (44), Woodruff (45), Millstein et al. (46)	Channels	Transaction cost, service quality, convenience, transaction risk, etc.
Mwencha et al. (47), Verhoef et al. (48)	Context	Physical conditions, urgency, etc.

#### 2.2.2. Consumer channel migration behavior in VAM

**Figure 3:**
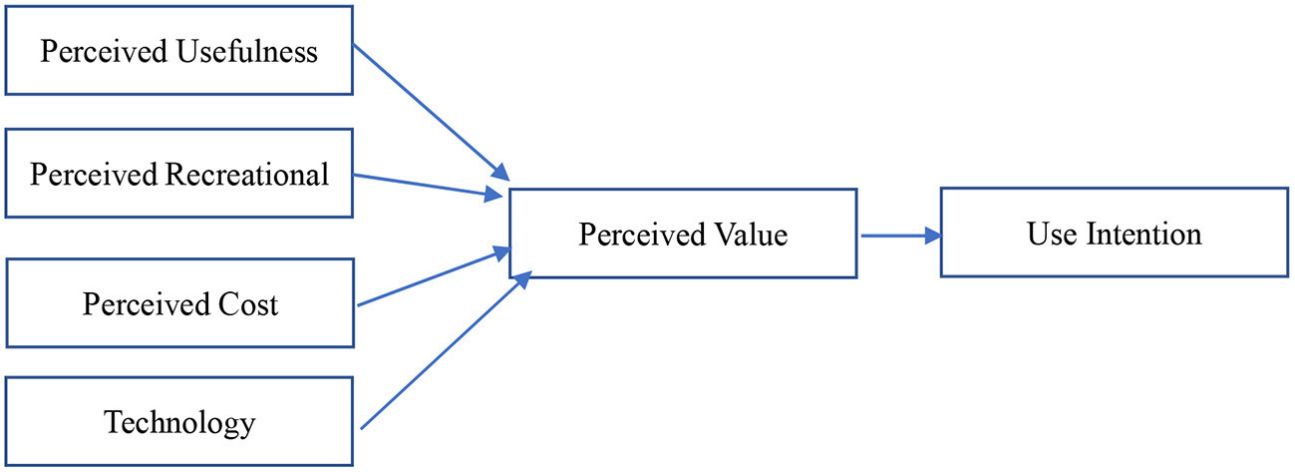
Perceived value acceptance model.

Compared with TAM, VAM holds that customer acceptance behavior is voluntary rather than passive. The VAM model is shown in Figure 3.

In VAM, consumers' perception of an external system is mainly divided into two aspects: income and payment. Perceived benefit consists of perceived usefulness and perceived entertainment, while perceived pay includes perceived cost and technical characteristics; these are combined to measure consumers' perceived value. Technical characteristics in the model refer to the cost of perceived payment that cannot be measured based on money, such as whether the system is easy to operate and the corresponding ease of use. Previous studies using VAM have used measurement items related to perceptual usability to measure technical characteristics. According to the VAM model, in the study of mobile internet users' intention to use a system, how users measure perceived return and perceived effort will directly affect perceived value, thus affecting their use intention (24, 49–54).

The VAM theoretical model has been widely used in the research on consumers' channel choice behavior under omnichannel conditions in recent years. Based on the TAM and VAM models, Cao and Li (49) explored the influence of consumers' perceived value when purchasing fresh products on their omnichannel acceptance attitude. Pentina and Hasty (55) used the VAM model and introduced a loyalty factor as a mediating variable to explore the factors that affect customers' channel switching in an omnichannel context. Perceived value, as a subjective evaluation of the value measurement of products or services, usually acts as a dependent variable or as a mediating variable in the purchase decision model (50). Existing research on the influencing factors of perceived value mainly focuses on antecedent variables and result variables, revealing a series of mechanisms of perceived value, which is of great significance to this study. Perceived value, as a subjective evaluation of consumers' benefits and costs, can effectively reflect consumers' supervisor preference, and thus explain and predict consumers' purchase intention for products or services (56). At the same time, perceived value has a positive impact on shopping satisfaction and loyalty. Perceived value can bring consumers a higher perception of interest, while high perceived value can make consumers actively recommend products to others and repurchase them (57, 58). Berman and Thelen (59) determined the important role of perceived value in consumer channel decision-making by constructing a mathematical model of consumer channel choice based on perceived value. At present, the research on the application of perceived value to consumers' channel choice mainly concerns the use of online channels. Van de Zande (60) verified that perceived value has a positive effect on consumers' intention to shop using online channels. Fang et al. (61), based on the expected utility theory, proposed that consumers' perceived value of channels has a positive impact on their intention to migrate to a channel for shopping.

This paper therefore takes perceived usefulness, perceived entertainment, and perceived cost as influencing factors, and further studies the influencing mechanism of consumers' channel migration behavior under normal epidemic prevention and control.

## 3. Hypotheses, model, and method

### 3.1. Hypotheses

#### 3.1.1. Factors affecting channel migration intention

Under normal epidemic prevention and control, the characteristics of different shopping channels and consumers' feelings will affect consumers' final choice of channels to a certain extent. Based on the PPM model, many scholars regard perceived usefulness of channels as a pulling factor in channel migration and have verified the influence of perceived usefulness on channel migration intention (16, 26). Li et al. (16) chose channel migration from offline to online as the research object, verifying that the perceived ease of use and perceived entertainment of online channels have a positive impact on consumers' intention to choose channels. Flavián et al. (29) highlighted that consumers' perceived usefulness and perceived entertainment have a positive effect on their intention-to-retain behavior across channels. This study therefore believes that consumers' perception of fresh product purchase channels will significantly affect their intention to migrate channels; thus, the following hypotheses are proposed:

- H1: Consumers' perceived usefulness of fresh product retailing channels has a positive effect on channel migration behavior.
- H2: Consumers' perceived entertainment of fresh product retailing channels has a positive effect on channel migration behavior.

Under normal epidemic prevention and control, consumers' choice of purchasing channels not only considers product quality and value but also monetary cost and convenience in the purchasing process. Consumers tend to choose purchasing channels with lower perceived cost. Haider et al. (22) explored channel migration from online to offline from the perspective of perceived cost and highlighted that consumers' perceived cost of online purchase channels positively affected their intention to use offline channels. At the same time, based on the PPM model, many believed that purchase cost has a boosting effect on consumers' intention to migrate to online channels (38, 62). Therefore, this paper argues that when consumers perceive higher costs in the process of purchasing fresh products, their intention of channel migration will be stronger. In summary, this study hypothesizes:

- H3: Consumers' perceived cost of fresh product retailing channels has a positive effect on channel migration behavior.

Under normal epidemic prevention and control, fresh retail enterprises pay more attention to channel integration to meet customers' shopping needs and experiences. Different integration forms constitute channel characteristics that further affect consumers' attitudes toward different shopping channels and channel decisions. The characteristics of online and offline goods, such as consistent categories and prices, positively affect consumers' attitude toward retail brands and positively affect consumers' intention to repurchase goods through online channels (39, 54, 63). Li et al. (44) found a positive impact of channel integration on consumers' cross-channel retention behavior by studying the purchasing behavior of cross-channel consumers. The channel characteristics brought about by channel integration further eliminate the barriers and differences between online and offline channels and affect consumers' channel decisions. Based on the above analysis, this study proposes the following hypothesis:

- H4: The channel characteristics of fresh product retailing channels negatively affect consumers' migration intention.

#### 3.1.2. Factors affecting perceived value

The perceived value theory put forward by Zeithaml (64) broke down perceived value into the two angles of gain and loss, and many have subsequently adopted this definition in research on consumer channel choice. At the same time, Ackermann and von Wangenheim (65) put forward the VAM theoretical model based on perceived value theory and verified that users' perceived usefulness, perceived entertainment, and perceived cost can affect perceived value from the two dimensions of perceived gain and loss. VAM has been applied in many different research fields since. Therefore, this paper proposes the following hypotheses:

- H5: Consumers' perceived usefulness of fresh product retailing channels positively affects perceived value.
- H6: Consumers' perceived entertainment of fresh product retailing channels positively affects perceived value.
- H7: Consumers' perceived cost of fresh product retailing channels negatively affects perceived value.

#### 3.1.3. Perceived value and migration intention

Perceived value is often introduced in studies on consumers' channel choice behavior. Shen et al. (66) found that the higher the perceived value, the stronger consumers' intention to buy online. Mwencha et al. (47) used the purchase of fresh agricultural products as an example and found that consumers' perceived profits positively affected their intention to buy. When consumers' perceived revenue is lower than their perceived pay, the overall perceived value of the channel will be lower, making channel migration behavior more likely to occur in channel decision-making. Acquila-Natale (18) conducted research based on consumers' online channel purchases and found that the higher consumers' perceived value of information collection channels, the stronger their intention to choose the same channel when they finally complete the purchase (45, 67); that is, consumers are less likely to have channel migration behavior. At the same time, the higher the perception of interests brought by purchasing channels to consumers, the easier it is for consumers to have cross-channel retention intention (68). Based on the above research, this paper proposes the following hypothesis:

- H8: Consumers' perceived value of fresh product retailing channels negatively affects their intention to migrate channels.

#### 3.1.4. The mediating effect between perceived value and migration intention

This study argues that the research on consumers' purchasing channels for fresh products should not only consider the influence of various factors on perceived value and channel migration intention but also the reduction of channel migration behavior on the basis of realizing perceived value (i.e., perceived value plays a mediating role between various influencing factors of the VAM model and channel migration intention). Kumar and Grisaffe (69) combined the TAM and VAM models in the research of online shopping intention and considered the mediating role of perceived value between perceived usefulness, perceived cost, and online shopping intention. In the research on channel switching intention and cross-channel retention intention, perceived value is also regarded as a mediating variable of perceived usefulness, perceived entertainment, and perceived risk, and the research has further explored the influencing mechanism (13, 19). Based on the VAM theoretical model and the above research, this paper proposes the following hypotheses (70):

- H9: Consumers' perceived value plays a mediating role between perceived usefulness and channel migration intention.
- H10: Consumers' perceived value plays a mediating role between perceived entertainment and channel migration intention.
- H11: Consumers' perceived value plays a mediating role between perceived cost and channel migration intention.

#### 3.2.5. The moderating effect of switching cost between perceived value and migration intention

For cross-channel consumers, switching cost has a certain limiting effect on channel migration (18). If the switching cost is high, consumers' intention to switch purchasing channels will be weakened. Chiu et al. (26) believed that consumers' intention to migrate to offline channels is affected by switching cost. Li et al. (44) put forward the moderating role of switching cost in perceived value and cross-channel retention intention. In research on the offline purchasing behavior of consumers, Salvietti et al. (11) put forward the moderating effect of switching cost on “anti-exhibition hall” purchasing behavior. Based on these studies, this paper proposes the following hypotheses:

- H12: Channel switching cost plays a moderating role between perceived value and channel migration intention.

### 3.2. Model and method

**Figure 4:**
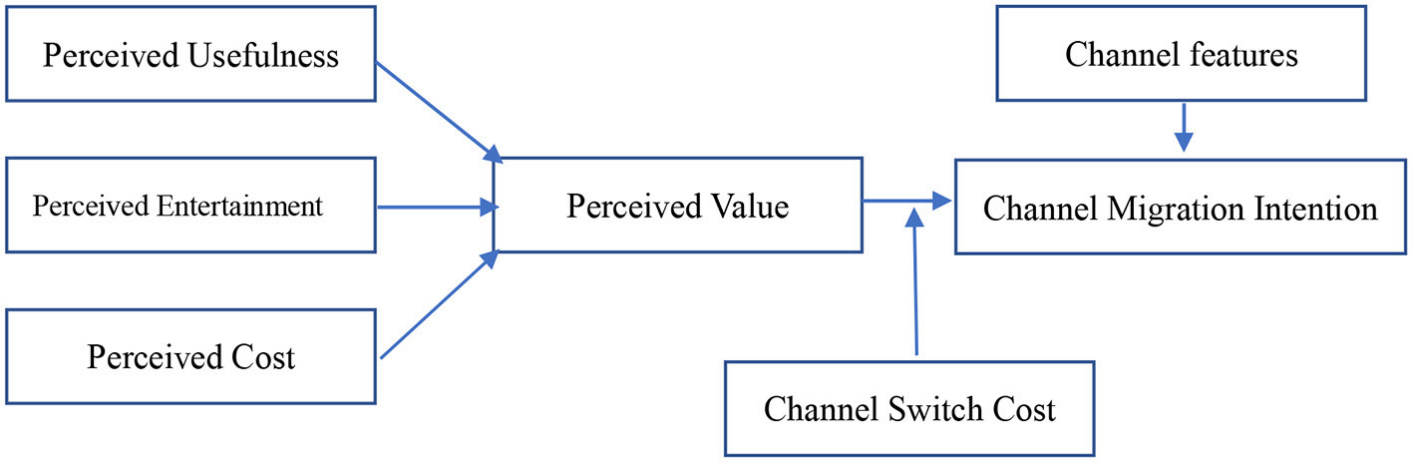
Research model.

Previous research has shown that the adoption of unified marketing methods by channel providers will affect consumers' channel choices (23). Gensler et al. (24) believed that the higher the value consumers perceive from a product or service, the stronger their intention to choose the product or service. This view is also applicable to the study of consumers' channel migration behavior. Verhoef and Donkers (51) used clothing products as an example to explore the mediating effect between the perception of perceived value in the process of consumers' purchase decision-making and the intention of stores to choose online shopping. Other researchers introduced perceived value into the research model of channel migration intention and thought that consumers' perceived usefulness and perceived entertainment of purchasing channels would have an impact on channel selection (25, 26). At the same time, VAM also puts forward the effects of perceived usefulness, perceived entertainment, and perceived cost on perceived value. Chiu et al. (26) also pointed out that switching cost plays a moderating role in perceived value and cross-channel retention intention. Therefore, combined with previous research theories and pre-investigation results, this paper introduces four independent variables: channel characteristics, perceived usefulness, perceived entertainment, and perceived cost. This paper also adds the mediating variable of perceived value between perceived usefulness, perceived entertainment, and perceived cost and channel migration intention, and adds the adjustment variable of channel switching cost between perceived value and channel migration intention to analyze the influencing mechanism of consumers' channel migration intention under normal epidemic prevention and control. The model is shown in Figure 4.

### 3.3. Measurements

#### 3.3.1. Perceived usefulness

Perceived usefulness is an important variable in the VAM theoretical model for fresh product purchase behavior, mainly reflected in the purchase channel to improve the degree of the consumer purchase effect. There are three items, which are measured based on the channels commonly used by consumers when purchasing fresh products, as shown in Table 2.

**Table 2:** Perceived usefulness measurement.

**Variable**	**Numbering**	**Item**	**References**
Perceived usefulness	PU1	This channel can let me get more abundant fresh product information	Byrd et al. (33), Goswami and Chouhan (34)
	PU2	This channel makes it more convenient for me to choose fresh products	
	PU3	This channel can meet my daily consumption demand for fresh products	

#### 3.3.2. Perceived recreational

Perceived usefulness in this study is reflected in the positive experience of consumers when they buy fresh products. The design of the measurement scale for perceived entertainment refers to the research and scales of previous studies and makes reasonable amendments to the scale according to the characteristics of fresh product purchase. There are four items in this variable, which are measured based on the channels commonly used by consumers when purchasing fresh products, as shown in Table 3.

**Table 3:** Perceived recreation measurement.

**Variable**	**Numbering**	**Item**	**References**
Perceived recreational	PR1	Using this channel to buy fresh products provides me with fun	Sweeney et al. (68), Zeithaml (64)
	PR2	Using this channel to buy fresh products is a leisure activity	
	PR3	When using this channel to purchase fresh products, time flies	
	PR4	I enjoy using this channel to buy fresh products	

#### 3.3.3. Perceived cost

There are four items in total, which are measured based on the channels commonly used by consumers when purchasing fresh products, as shown in Table 4.

**Table 4:** Perceived cost measurement.

**Variable**	**Numbering**	**Item**	**References**
Perceived cost	PC1	I need to spend more time shopping for fresh products	Gupta et al. (38), Yang et al. (19)
	PC2	I need to spend more energy buying fresh products	
	PC3	I need to spend a lot of network traffic or transportation costs to buy fresh products	
	PC4	This channel is very affordable to buy fresh products	

#### 3.3.4. Channel characteristics

The degree of integration of online and offline retail channels of fresh products reflects the possibility of providing consumers with a seamless shopping experience. In this paper, the channel characteristics under normal epidemic prevention and control are divided into four dimensions: product information, product price, preferential form, and inventory visibility. Based on the research and scales of previous studies, there are four items after modification according to specific contents, as shown in Table 5.

**Table 5:** Channel characteristics measurement under normalized epidemic prevention and control.

**Variable**	**Numbering**	**Item**	**References**
Channel characteristics	OCF1	The commodity information of fresh products is consistent online and offline channels	Li et al. (44), Trampe et al. (39)
	OCF2	The pricing of fresh products is consistent online and offline channels	
	OCF3	Preferential forms such as points are consistent online and offline channels	
	OCF4	Fresh product inventory can be inquired online and offline channels	

#### 3.3.5. Perceived value

Perceived value includes functional value, emotional value, price value, and social value. The scale design of perceived value uses the definition of Sweeney and other scholars. There are four items for the four dimensions of perceived value, which are measured based on the channels commonly used by consumers when purchasing fresh products. The revised scale combined with the research content is shown in Table 6.

**Table 6:** Perceived value measurement.

**Variable**	**Numbering**	**Item**	**References**
Perceived value	PV1	The fresh products purchased through this channel are of good quality	Sweeney and Soutar (68), Mwencha et al. (47)
	PV2	The channel is well used	
	PV3	Using this channel to buy fresh products is cost-effective	
	PV4	Using this channel to buy fresh products is a wise choice	

#### 3.3.6. Channel switching cost

The conversion cost can be divided into time cost, energy cost, monetary cost, and psychological cost. Aiming at these four dimensions, this study refers to the measurement scales of previous studies and modifies the research content. Based on the conversion cost of fresh product purchase channels, there are five items, as shown in Table 7.

**Table 7:** Channel switching cost measurement.

**Variable**	**Numbering**	**Item**	**References**
Channel switching cost	CSC1	Switching to a new shopping channel can take me a lot of time	Gupta et al. (38), Zhang et al. (57), Dong and Lu (50)
	CSC2	Switching to a new shopping channel takes a lot of energy	
	CSC3	Switching to a new shopping channel will cost me more money	
	CSC4	It would be inconvenient for me to switch to a new shopping channel	
	CSC5	I'm worried that I won't get the same service as the old one after switching to a new shopping channel	

#### 3.3.7. Channel switching intention

Channel switching behavior and channel free-riding behavior together constitute consumers' channel migration behavior (71). The scale design refers to previous studies and designs a fresh product purchase channel migration intention scale with three items covering two dimensions and four paths of channel migration, as shown in Table 8.

**Table 8:** Channel migration intention measurement.

**Variable**	**Numbering**	**Item**	**References**
Channel switching intention	CMI1	When buying fresh products again, I plan to change the purchase channel	Gupta et al. (38), Ansari et al. (63)
	CMI2	When I buy fresh products again, I will learn about the products in one retailer's offline store and make purchases in another retailer's online store	
	CMI3	When I buy fresh products again, I will learn about the products in one retailer's online store and make purchases in another retailer's offline physical store	

## 4. Data and analysis

### 4.1. Questionnaire and sample

In this study, questionnaires were distributed online, made using the Questionnaire Star platform, and recovered through WeChat, Weibo, Douban Group, and the Questionnaire Star filling community. This group has a wide age coverage, mainly between 18 and 30 years old, and has a high network intake, which meets the research needs of this study. A total of 322 questionnaires were collected in this questionnaire survey, and 292 valid questionnaires were obtained after eliminating invalid questionnaires.

### 4.2. Descriptive statistics

A total of 292 valid samples were collected in this survey. SPSS 21.0 was used to analyze the basic information of the sample; the results are shown in Table 9.

**Table 9:** Sample basic information.

**Attribute**	**Characteristic**	**Quantity**	**Proportion**
Gender	Male	126	43.2
	Female	166	56.8
Age	Below 18	3	1.0
	18–25 years old	181	62.0
	26–30 years old	72	24.7
	31–40 years old	25	8.6
	Over 40	11	3.8
Academics	Middle school	2	0.7
	Secondary/high school	10	3.4
	College	11	3.8
	Undergraduate	239	81.8
	Master and above	30	10.3
Shopping channels	Online	119	40.8
	Offline	173	59.2

As can be seen from Table 9, in terms of gender, there were more women in this questionnaire survey, but it was relatively balanced. In terms of age, the survey population was mainly 18–30 years old, accounting for 86.7% of the total sample. In terms of educational background, the respondents were highly educated, mainly with a bachelor's degree or above, accounting for 92.1%. In terms of common purchasing channels for fresh products, 59.2% of the respondents often used offline channels to purchase fresh products.

The data of other scales was analyzed using SPSS 21.0, with a total of seven variables and 27 measurement items. The data for each measurement item are shown in Table 10.

**Table 10:** Basic information of each item data.

**Item**	**Min**	**Max**	**Avg**	**SD**
PU1	1	5	4.25	0.643
PU2	1	5	4.19	0.770
PU3	1	5	4.14	0.678
PR1	1	5	3.57	0.708
PR2	1	5	3.57	0.795
PR3	1	5	3.72	0.747
PR4	1	5	3.79	0.694
PC1	1	5	3.38	0.847
PC2	1	5	3.27	0.833
PC3	1	5	3.20	0.822
PC4	1	5	2.44	0.586
OCF1	1	5	3.29	0.908
OCF2	1	5	2.93	0.944
OCF3	1	5	3.00	0.984
OCF4	1	5	3.30	0.938
PV1	1	5	3.89	0.673
PV2	1	5	3.92	0.708
PV3	2	5	3.98	0.493
PV4	1	5	3.89	0.662
CSC1	1	5	3.56	0.977
CSC2	1	5	3.55	0.999
CSC3	1	5	3.23	0.984
CSC4	1	5	3.43	1.022
CSC5	1	5	3.79	0.995
CMI1	1	5	2.55	0.795
CMI2	1	5	2.69	0.866
CMI3	1	5	2.74	0.930

### 4.3. Reliability

Reliability refers to the consistency of data collected by a scale, and it is an important standard by which to determine whether the measurement results are reliable. Generally, scholars believe that the reliability of a scale should be at least 0.6 and the reliability is acceptable between 0.7 and 0.8. If it is >0.8, the reliability is good. In this study, SPSS 21.0 was used to analyze the reliability of the scale as a whole and each dimension scale (see Table 11).

**Table 11:** Results of reliability analysis.

**Variable**	**Cronbach's alpha**	**Items**
Perceived usefulness	0.799	3
Perceived recreational	0.727	4
Perceived cost	0.819	4
Channel characteristics	0.815	4
Perceived value	0.754	4
Channel switching cost	0.833	5
Channel switching intention	0.825	3

As shown in Table 11, the Cronbach's alpha coefficient of the overall scale of 27 items in this study was 0.708; therefore, the reliability was in an acceptable range. Cronbach's alpha coefficients of perceived usefulness, perceived entertainment, and perceived value were all between 0.7 and 0.8, and so were still in the acceptable reliability range. The Cronbach's alpha coefficient of perceived cost, channel characteristics, channel switching cost, and channel migration intention was >0.8, which shows that the reliability of the measurement results for these four dimensions was good. Generally speaking, the reliability of this study scale was within an acceptable range.

### 4.4. Validity

Before confirmatory factor analysis, Kaiser–Meyer–Olkin (KMO) tests and Bartlett spherical tests were carried out on the scale (see Table 12).

**Table 12:** KMO and Bartlett tests.

Kaiser–Meyer-Olkin measure of sampling adequacy		**0.750**
Bartlett tests	The approximate chi-square	3,162.262
	df	351
	Sig.	0.000

The KMO value of this research scale was 0.750, which is in the range 0.7–0.8, indicating that this scale has relatively good validity. Bartlett's spherical test returned a significance of < 0.001, indicating that there was a strong correlation among the variables. It can be seen that this research scale is suitable for factor analysis. Confirmatory factor analysis on 27 measurement items of perceived usefulness, perceived entertainment, perceived cost, channel characteristics, perceived value, channel switching cost, and channel migration intention was carried out using SPSS, and the results are shown in Tables 13, 14.

**Table 13:** Factor loading.

**Variable**	**Items**	**C.R**.	**AVE**
Perceived usefulness	PU1	0.813	0.699
	PU2		
	PU3		
Perceived recreational	PR1	0.732	0.601
	PR2		
	PR3		
	PR4		
Perceived cost	PC1	0.795	0.632
	PC2		
	PC3		
	PC4		
Channel characteristics	OCF1	0.741	0.618
	OCF2		
	OCF3		
	OCF4		
Perceived value	PV1	0.788	0.676
	PV2		
	PV3		
	PV4		
Channel switching cost	CSC1	0.769	0.653
	CSC2		
	CSC3		
	CSC4		
	CSC5		
Channel migration intention	CMI1	0.851	0.702
	CMI2		
	CMI3		

**Table 14:** Model fitting check.

**Model fitting index**	**Valuation**	**Statistics**
CMIN	/	462.203
DF	/	298
CMIN/DF	< 2	1.551
RMSEA	< 0.08	0.044
SRMR	< 0.08	0.034
CFI	>0.9	0.944
GFI	>0.9	0.902
TLI	>0.9	0.934
IFI	>0.9	0.945

The standardized factor load coefficients of each item and latent variable were in a reasonable range, ranging from 0.732 to 0.851, and the average variance extracted (AVE) was between 0.601 and 0.702, which indicates that the data can be considered independent. Therefore, we can continue to analyze the specific data of indicators, such as model fitting degree.

The ratio of chi-square to degree of freedom was 1.551, reaching the standard of < 2. The fitting indexes GFI, CFI, TLI, and IFI were all >0.9, and RMSEA was 0.044, SRMR was 0.034, all of which reached the standard of < 0.08. Therefore, it can be considered that this research model has a good degree of fit.

### 4.5. Model analysis

#### 4.5.1. Correlation

After verifying the fitting degree of the model, SPSS 21.0 was used to analyze the correlation of the model research variables; the correlation coefficients are listed in Table 15.

**Table 15:** Correlation coefficients.

**Variable**	**1**	**2**	**3**	**4**	**5**	**6**	**7**
PU	1						
PR	0.217^***^	1					
PC	−0.067	0.119^*^	1				
CC	0.101	0.296^***^	0.038	1			
PV	0.366^***^	0.329^***^	−0.148^*^	0.186^**^	1		
CSC	0.034	−0.034	−0.003	0.068	−0.043	1	
CMI	−0.189^**^	−0.172^**^	0.241^***^	−0.178^**^	−0.251^***^	0.075	1

*N* = 292.

^*^P < 0.05.

^**^P < 0.01.

^***^P < 0.001.

Perceived entertainment was positively correlated with perceived usefulness (*r* = 0.217, *P* < 0.001), perceived cost (*r* = 0.119, *P* = 0.043), and channel characteristics (*r* = 0.296, *P* < 0.001). Perceived value was positively correlated with perceived usefulness (*r* = 0.366, *P* < 0.001), perceived entertainment (*r* = 0.329, *P* < 0.001), and channel characteristics (*r* = 0.186, *P* = 0.001), and was negatively correlated with perceived cost (r = −0.148, *P* = 0.011); Channel migration intention was positively correlated with perceived cost (*r* = 0.241, *P* < 0.001) and negatively correlated with perceived usefulness (*r* = −0.189, *P* = 0.001), perceived entertainment (*r* = −0.172, *P* = 0.003), channel characteristics (*r* = −0.178, *P* = 0.002), and perceived value (*r* = −0.251, *P* < 0.001).

In summary, perceived usefulness, perceived entertainment, perceived cost, and channel characteristics were found to be significantly correlated with perceived value and channel migration intention, and there was also a significant correlation between perceived value and channel migration intention.

#### 4.5.2. Regression

Linear regression in SPSS 21.0 was used to analyze the relationship between perceived value and channel migration intention, which has four influencing factors: perceived usefulness, perceived entertainment, perceived cost, and channel characteristics. All the VIF of the variables were analyzed, with resultant values lower than 3, which indicates an acceptable result. The basic information of samples were used as control variables in the model, and the final analysis results are presented in Table 16.

**Table 16:** Regression.

**Variable**	**Perceived value**		**Channel migration intention**			
	**M1**	**M2**	**M3**	**M4**	**M5**	**M6**
Intercept	3.888^***^	2.322^***^	2.204^***^	2.879^***^	3.655^***^	3.342^***^
Gender	−0.049	−0.038	0.263^**^	0.240^**^	0.244^**^	0.232^**^
Age	0.001	−0.045	−0.064	−0.031	−0.064	−0.040
Academics	0.021	0.011	0.026	0.021	0.034	0.023
Purchased Channel	0.014	0.040	0.065	0.045	0.071	0.053
PU		0.246^***^		−0.158^*^		−0.109
PR		0.234^***^		−0.160^*^		−0.113
PC		−0.123^**^		0.291^***^		0.266^***^
CC		0.060		−0.127^*^		−0.115^*^
PV					−0.373^***^	−0.199^*^
*R* ^2^	0.003	0.238	0.047	0.164	0.106	0.177
Adjust *R*^2^	−0.011	0.217	0.034	0.141	0.090	0.151

*N* = 292.

^*^P < 0.05.

^**^P < 0.01.

^***^P < 0.001.

As shown in M2 in Tables 4–8, perceived usefulness and perceived entertainment have a significant positive impact on perceived value, while perceived cost has a significant negative impact on perceived value; therefore, hypotheses H5, H6, and H7 are verified. At the same time, it can be seen from M5 that perceived value has a significant negative impact on channel migration intention; therefore, H8 also holds.

To analyze the mediating effect of perceived value, the first step was to test the main effect. As M4 shows, after controlling for the influence of gender, age, and other variables, perceived usefulness, perceived entertainment, and channel characteristics have positive effects on channel migration intention, while perceived cost has significant negative effects on channel migration intention. Therefore, H1, H2, H3, and H4 hold true.

The influence of the independent variables of perceived usefulness, perceived entertainment, perceived cost, and channel characteristics on the mediator variable of perceived value was tested. After controlling for the influence of gender, age, and other variables, as shown in M2, perceived usefulness and perceived entertainment have a significant positive impact on perceived value, perceived cost has a significant negative impact on perceived value, and channel characteristics have no significant impact on perceived value.

The influence of perceived value of mediating variables on the intention of dependent variables to migrate through channels was then tested. After controlling for the influence of gender, age, and other variables, as shown by M6, perceived value has a negative effect on channel migration intention. Currently, perceived usefulness and perceived entertainment have significant effects on channel migration intention, which no longer significantly affects channel migration intention (i.e., perceived value plays a complete mediating role). At the same time, perceived cost still has a significant negative impact on channel migration intention, but compared with M4, the regression coefficient of perceived cost decreases from 0.291 to 0.266 (i.e., perceived value plays a partial mediating role). Therefore, hypotheses H9, H10, and H11 hold true.

Analyzing the adjustment effect of channel switching cost (see M7 and M8 in Table 17), the interaction between perceived value and channel switching cost has no significant impact on channel migration intention (i.e., there is no moderating effect); thus, H12 is not valid.

**Table 17:** Regression of moderating effect analysis.

**Variable**	**Channel migration intention**				
	**M3**	**M4**	**M6**	**M7**	**M8**
**Control variable**					
Gender	0.263^**^	0.240^**^	0.232^**^	0.228^*^	0.227^*^
Age	−0.064	−0.031	−0.040	−0.046	−0.041
Academic	0.026	0.021	0.023	0.019	0.006
Purchased channel	0.065	0.045	0.053	0.049	0.058
**Independent variable**					
PU		−0.158^*^	−0.109	−0.114	−0.122
PR		−0.160^*^	−0.113	−0.107	−0.108
PC		0.291^***^	0.266^***^	0.267^***^	0.259^***^
CC		−0.127^*^	−0.115^*^	−0.122^*^	−0.120^*^
**Mediating variable**					
PV			−0.199^*^	−0.192^*^	−0.201^*^
**Moderating variable**					
CSC				0.078	0.081
**Mediation, adjusting interaction items**					
Perceived value × channel switching cost					−0.176
*R* ^2^	0.047	0.164	0.177	0.183	0.192
Adjust *R*^2^	0.034	0.141	0.151	0.154	0.160

*N* = 292.

^*^P < 0.05.

^**^P < 0.01.

^***^P < 0.001.

Based on the above data presentation and analysis, the results of the hypothesis tests are summarized in Table 18.

**Table 18:** Research hypothesis testing.

**No**.	**Hypotheses**	**Result**
H1	Consumers' perceived usefulness of fresh products retailing channels have positive effects on channel migration behavior	Support
H2	Consumers' perceived entertainment of fresh products retailing channels have positive effects on channel migration behavior	Support
H3	Consumers' perceived cost of fresh products retailing channels have positive effects on the channel migration behavior	Support
H4	The channel characteristics of fresh products retailing channels negatively affect consumers' migration intention	Support
H5	Consumers' perceived usefulness of fresh products retailing channels positively affects perceived value	Support
H6	Consumers' perceived entertainment of fresh products retailing channels positively affects perceived value	Support
H7	Consumers' perceived cost of fresh products retailing channels negatively affects perceived value	Support
H8	Consumers' perceived value of fresh products retailing channels negatively affects their intention to migrate channels	Support
H9	Consumers' perceived value plays a mediating role between perceived usefulness and channel migration intention	Support
H10	Consumers' perceived value plays a mediating role between perceived entertainment and channel migration intention	Support
H11	Consumers' perceived value plays a mediating role between perceived cost and channel migration intention	Support
H12	The channel switching cost plays a moderating role between perceived value and channel migration intention	Not support

## 5. Discussion and conclusions

### 5.1. Discussion

In this study, we proposed a model to determine the influencing factors of consumer channel migration behavior during the COVID-19 pandemic. Referring to Table 18, we discuss the results in the following groups.

First, from H1, H2, H3, H5, H6, and H7, we see that consumers' perceived usefulness, perceived entertainment, and perceived cost of fresh product retailing channels have a positive effect on channel migration behavior, which is consistent with previous theoretical studies (16, 26, 38, 62). The difference between our findings and previous studies lies in the research context, as we know that the COVID-19 pandemic has had a significant effect on daily life and purchasing behavior. In the context of this pandemic, we have confirmed that consumers will probably change to other retailers when the usefulness, entertainment, and cost meet their expectation for purchasing fresh products. Retailers should also enhance the shopping experience of consumers during the pandemic.

Second, from H4 and H8, we have proved that channel characteristics and perceived value will negatively affect the intention to migrate channels, which is contrary to previous studies (39, 44, 63). Channel characteristics have versatile features, such as channel structure and supply chain mode, which affect consumer behaviors in different ways. The perceived value comes from expectations and experience. Unless the experience is enhanced, the perceived value will increase. This finding confirms that experience is a critical factor in channel migration behavior.

Third, from H9, H10, H11, and H12, the mediating effect of consumer perceived value on the perceived usefulness, perceived entertainment, and perceived cost of the channel migration intention is confirmed, which is consistent with previous studies (13, 19). Retailers should increase consumer's perceived value to maximize their profit. In H12, the result is different from previous studies (18, 26). The reason for this issue is mainly the type of fresh products. In previous studies, the consumer was focusing on products except for fresh products. From this result, retailers should try to keep their products fresh and provide consumers with a high-level shopping experience during sale.

### 5.2. Conclusions

Under normal epidemic prevention and control, this paper studies the relationship between perceived usefulness, perceived entertainment, perceived cost, channel characteristics of normal epidemic prevention and control, perceived value, and channel migration intention in fresh product purchase based on VAM. Through the design and distribution of the scale and the follow-up empirical analysis, this study draws the following conclusions.

First, in the context of normalized epidemic prevention and control, the development of the epidemic situation and the implementation of epidemic prevention plans and measures continue to affect the consumption behavioral intention of Chinese residents, thus changing consumer behavior and decision-making. From the perspective of consumption, the epidemic has had a significant impact on residents' consumption expenditure and the frequency of going out to spend money. During the outbreak period, residents' consumption expenditure decreased due to the lockdown and home isolation. At that time, experts and scholars at home and abroad believed that there would be “retaliatory consumption” after the resumption of work. However, with the continuous implementation of normalized epidemic prevention, continuous retaliatory consumption did not appear, and consumption expenditure neither decreased nor increased. A large part of the reason is that online consumption effectively compensated for offline consumption. From the perspective of consumption, it is frequently reported that imported goods carry COVID-19 disclosure information during the normalized epidemic prevention period, which makes domestic residents pay more attention to the safety of domestic and imported goods and their packaging. From the perspective of consumption patterns, online shopping provides consumers with contactless distribution services to meet the needs of residents to avoid contact with the outside world and protect their lives. It has become a common mode under normal epidemic prevention, especially in some places where there are still restrictions on entry and exit. Online shopping meets more of the needs of consumers. Furthermore, emerging information technology is booming, and online consumption is pushed more accurately through technologies such as big data, especially short videos and live broadcasts, which fully mobilize residents' intention to consume. From the perspective of the consumption concept, the outbreak of the epidemic and staying at home in the long term forced residents to generate more consumption demand related to health and green living (e.g., the demand for health care products, sports equipment, and organic food is rising), and this rising trend will continue under the normal epidemic prevention campaign.

Second, when consumers purchase fresh products, the perception of channels impacts their behavior. The stronger the perception of entertainment, the higher the perceived value of channels, while perceived cost has a negative impact on perceived value. Perceived usefulness and perceived entertainment are consumers' perceived benefits of purchasing channels. The more convenient and useful the channels are, the stronger the channel experience is, which can bring higher channel perceived value. Perceived cost reflects consumers' perceived contribution in channel selection. The higher the perceived cost, the lower the perceived value of the channels.

Third, perceived usefulness, perceived entertainment, channel characteristics, and perceived value have significant negative effects on channel migration intention, while perceived cost has significant positive effects on channel migration intention. It can be seen from this that consumers attach great importance to the convenience and experience of channels when choosing channels. When common purchasing channels meet consumers' needs for perceived usefulness and perceived entertainment, consumers are more inclined to use these channels, and their intention to migrate channels is weak. At the same time, as consumers' perceived expenditure in purchasing decisions, perceived cost also has an impact on consumers' choices. The channel characteristics reflect the degree of channel integration. When the degree of channel integration is higher, it can provide consumers with a seamless consumption experience, meet consumers' personalized channel preferences, realize customer retention, and negatively affect consumers' channel migration behavior of switching retailers when shopping across channels.

Fourth, perceived value plays a complete mediating role between perceived usefulness, perceived entertainment, and channel migration intention, and partially mediates between perceived cost and channel migration intention. This also verifies the applicability of VAM for consumers' purchasing channels. Whether the purchasing channel of fresh products is convenient and whether the experience is good mainly affects channel migration by affecting the overall perception of consumers. The perceived cost not only has an impact on the overall perception of the purchase channel but also directly affects channel migration. When using a certain channel requires spending more nonmonetary costs (such as time or energy) or monetary costs, consumers' intention to migrate channels will be stronger.

Finally, the moderating effect of channel switching cost on perceived value and channel migration intention is not significant. Although in previous research, the higher the channel switching cost, the stronger the consumers' intention to retain across channels, channel migration behavior is different from cross-channel retention intention. The premise of cross-channel retention intention is that consumers choose the same retailer when dynamically changing channels in the two stages of information collection and purchase. However, besides considering the channel transformation of consumers, channel migration also needs to consider the change in consumers' choice of retailers. Therefore, the final adjustment effect of channel conversion cost is not clear.

### 5.3. Implications

#### 5.3.1. Theoretical implications

This paper examines the change in consumers' channel migration behavior under the pandemic in an empirical study and explores the factors that affect migration behavior. In the theoretical sphere, this paper extends the literature on consumer channel behavior and its research scope. We discuss the effect of channel characteristics on migration behavior and find that channel characteristics have a significant impact on channel migration. Previously, many studies investigated the influencing factors of channel behavior under a certain channel context or with a certain characteristic as the endogenous variable, and the effect of the channel characteristics received little attention (5, 18, 19, 21, 54). The results of this paper expand on previous studies. Retailers first need to have multiple purchase channels, and at the same time, they need to integrate existing channels, such as selling the same products online and offline at the same price, ensuring that inventory is visible, and providing various ways of picking up and returning goods, such that each channel is no longer independent. Consumers can meet many retailers and use different retail channels in the process of purchase decision-making. If the retail channels are independent of each other, they will easily eat into each other, which will weaken their overall competitiveness and is not conducive to the occurrence of cross-channel retention behavior. Through full channel integration, all channels can provide customers with a seamless shopping experience in the process of conversion by exerting synergy, this reducing consumers' channel migration behavior.

#### 5.3.2. Practical implications

With the continuous updating and iteration of information technology, consumer channel migration is becoming more inevitable. Therefore, retailers need to understand the mechanism behind it and develop corresponding strategies to reduce the channel migration of consumers and realize customer retention. According to the research conclusions, this paper puts forward the following suggestions for fresh product retailers.

First, retailers should improve channel service quality and consumers' perceived value. The results show that consumers' perceived usefulness, perceived entertainment, and perceived cost of channels are all related to perceived value, and the higher the perceived value, the weaker consumers' intention to migrate channels. Therefore, improving the service quality of retail channels plays an important role in enhancing users' awareness of the channels. Retailers should provide consumers with more convenient channel services and improve consumers' perception of purchasing channels in this way, such as by providing more comprehensive information for goods sold online and improving the training system to improve the service level of shopping guides in offline stores. At the same time, retailers should provide consumers with channel services with a stronger experience to improve consumers' entertainment perception of purchasing channels, such as improving store furnishings and providing consumers with a good shopping environment and atmosphere.

Second, retailers should reduce consumers' perceived cost to reduce channel migration behavior. Perceived cost not only affects consumers' perceived value but also has a positive impact on consumers' channel migration behavior. In the process of consumers' purchase decision-making, the time, energy, and money spent using a certain channel will directly affect the intention of channel conversion. Therefore, retailers need to ensure that channels provide services to consumers in a timely manner, such as improving the response speed of online channels, improving logistics efficiency, reasonably setting up offline store furnishings to reduce waiting time in line, and reducing the energy consumers need to spend to purchase goods.

### 5.4. Limitations and future work

Due to constraints in time and financial budget, this paper concentrated efforts on the young population only. In the future, this study might expand to include other ages, increase the sample size, and compare the findings. Further, a longitudinal study is advised for the future. Variables such as channel switching cost and perceived cost can be combined in a future study. Moreover, the channel switching cost and perceived cost may have some interaction, the two variables could be combined, and the moderating or mediating effect of the cost could be determined in future work.

## Data availability statement

The original contributions presented in the study are included in the article/supplementary material, further inquiries can be directed to the corresponding author/s.

## Author contributions

DW contributed to conception, design of the study, and wrote the first draft of the manuscript. WC and XZ organized the database. XW performed the statistical analysis. All authors contributed to manuscript revision, read, and approved the submitted version.

## Footnotes

^1^ https://data.stats.gov.cn/easyquery.htm?cn=C01
